# Reinforcement Q-Learning Control With Reward Shaping Function for Swing Phase Control in a Semi-active Prosthetic Knee

**DOI:** 10.3389/fnbot.2020.565702

**Published:** 2020-11-26

**Authors:** Yonatan Hutabarat, Kittipong Ekkachai, Mitsuhiro Hayashibe, Waree Kongprawechnon

**Affiliations:** ^1^Neuro-Robotics Laboratory, Graduate School of Biomedical Engineering, Tohoku University, Sendai, Japan; ^2^Smart Machine and Mixed Reality (SMR) Laboratory, National Electronics and Computer Technology Center (NECTEC), Pathum Thani, Thailand; ^3^Department of Robotics, Graduate School of Engineering, Tohoku University, Sendai, Japan; ^4^School of Information Computer and Communication Technology (ICT), Sirindhorn International Institute of Technology (SIIT), Thammasat University, Pathum Thani, Thailand

**Keywords:** reinforcement learning, reward shaping, Q-learning, semi-active prosthetic knee, magnetorhelogical damper

## Abstract

In this study, we investigated a control algorithm for a semi-active prosthetic knee based on reinforcement learning (RL). Model-free reinforcement Q-learning control with a reward shaping function was proposed as the voltage controller of a magnetorheological damper based on the prosthetic knee. The reward function was designed as a function of the performance index that accounts for the trajectory of the subject-specific knee angle. We compared our proposed reward function to a conventional single reward function under the same random initialization of a *Q*-matrix. We trained this control algorithm to adapt to several walking speed datasets under one control policy and subsequently compared its performance with that of other control algorithms. The results showed that our proposed reward function performed better than the conventional single reward function in terms of the normalized root mean squared error and also showed a faster convergence trend. Furthermore, our control strategy converged within our desired performance index and could adapt to several walking speeds. Our proposed control structure has also an overall better performance compared to user-adaptive control, while some of its walking speeds performed better than the neural network predictive control from existing studies.

## 1. Introduction

The knee joint enables one to perform basic movements, such as walking. The loss of this function such as in the case of transfemoral amputation could severely restrict movements. The lower limb prosthetic system, which comprises either the prosthetic knee, leg, or foot, could replace the function of the biological knee. Generally, the prosthetic knee is divided into two categories, that is, a mechanical-based control and microprocessor controlled. Reportedly, using the microprocessor-controlled prosthetic knee can improve the lower extremity joint kinetics symmetry, gait, and balance, as well as reduce the frequency of stumbling and falling, compared to using the mechanical or passive knee (Hafner et al., 2007; Kaufman et al., 2007, 2012; Sawers and Hafner, 2013).

Generally, the actuator in a microprocessor-controlled prosthetic knee can be divided into two categories: semi-active and active mechanisms. An active mechanism can generate a net positive force. Several institutions have been developing the active knee for research and development purposes (Hoover et al., 2012; Lawson et al., 2014; Flynn et al., 2015). However, owing to the high requirements of the actuation unit as well as the control system in terms of design and cost (Windrich et al., 2016), there has been only a few of the commercialized product in this category, such as the Power Knee (Össur, Iceland)^1^.

On the contrary, a semi-active mechanism or also called a variable-damping mechanism could only manipulate damping force. Magnetorheological (MR) damper is one of the examples that utilize this function by manipulating the strength of the magnetic field, which is applied to magnetic particles in a carrier fluid. The advantages of using this system are the rapid response and low power consumption, among others (Şahin et al., 2010). Therefore, from the cost-effective and functionality point of view, a semi-active prosthetic knee is still more favorable for the end user compared to the active mechanism. Consequently, in this study we focused on the control of prosthetic knee devices with a semi-active mechanism in a swing phase of the gait cycle.

Many studies on the prosthetic knee control algorithm have been conducted. The user-adaptive control as investigated in Herr and Wilkenfeld (2003) is an example of an adaptive control that applied the MR damper-based prosthetic knee. The underlying principle of this controller is to change the necessary damping required in each state if the biological knee trajectory deviated based on the information of the local sensing device. A finite state machine-based controller is often found in the powered knee (Wen et al., 2017). This controller is programmed to provide a control output of the current state machine obtained from specific rules based on varying sensing information. There has been an attempt to unify the prosthetic controller through discrete Fourier transform virtual constraints (Quintero et al., 2017). Furthermore, EMG-based control has been investigated in several studies, such as in Hoover et al. (2012). While this control has promising results, its application is limited to those who still have intact muscle function on the amputation site.

Several studies have tried to apply machine learning algorithm to control prosthetic (Ekkachai and Nilkhamhang, 2016; Wen et al., 2017, 2019). Neural network predictive control (NNPC) was employed as a control structure for the swing phase in the prosthetic knee (Ekkachai and Nilkhamhang, 2016). The swing phase model was constructed following a feed-forward neural network structure in which the input and the output were the knee angle, control voltage, and prediction of future knee angle. However, it requires an off-line training process to find weight and bias of neural network. Thus, when neural network has been trained, it will not have a mechanism to adapt the model. This raises a need of online learning model that could adapt if users change walking pattern due to weight change or using different costume.

An adaptive dynamic programming was employed in each state of walking for automatic tuning of the knee joint impedance parameter (Wen et al., 2017) and further improved into an online reinforcement learning (RL)-based control to tune a total of 12 impedance parameters of robotic knee prosthesis (Wen et al., 2019). Although it has shown potential outcome for human-prosthesis control tuning in a real time setting, the proposed algorithm is needed to tune a total 12 impedance parameters for 4 phases of walking. This is understandable since it was applied to powered prosthetic knee (Wen et al., 2019).

In this study, we investigated a model-free Q-learning control algorithm with a reward shaping function as the swing phase control in the MR damper-based prosthetic knee. A model-free algorithm could simplify the need for prior information, thus it could be implemented to different subjects effectively. We found that our proposed reward shaping function leads to better performance in terms of normalized root mean squared error and also showed a faster convergence trend compared to a conventional single reward function. Our proposed approach was also compared to user-adaptive control and NNPC from existing studies, which resulted in overall better performance across tested walking speeds.

The rest of this paper is organized as follows. Section 2 describes the specific MR damper system, double pendulum model as the environment, and the dataset that we used, as well as the details on Q-learning control. Section 3 presents the simulation and results. Finally, Section 4 discusses the algorithm comparison, the limitations, and the future works of this study.

## 2. Materials and Methods

In this section, we introduce the system, the environment model, and the RL algorithm we designed in this study. MR damper is defined as the system, that is, the main actuator to be controlled. Meanwhile, the environment is defined as the application where the system was used; in this case, a simple double pendulum model was used as the simulated environment to perform swing phase on a gait cycle. Section 2.1 covers a brief descriptions on the system and environment as well as dataset used in this study. Further, Q-learning algorithm designed for this study is discussed in detail in section 2.2.

### 2.1. System, Environment Model, Dataset

#### 2.1.1. System Description

**Figure 1:**
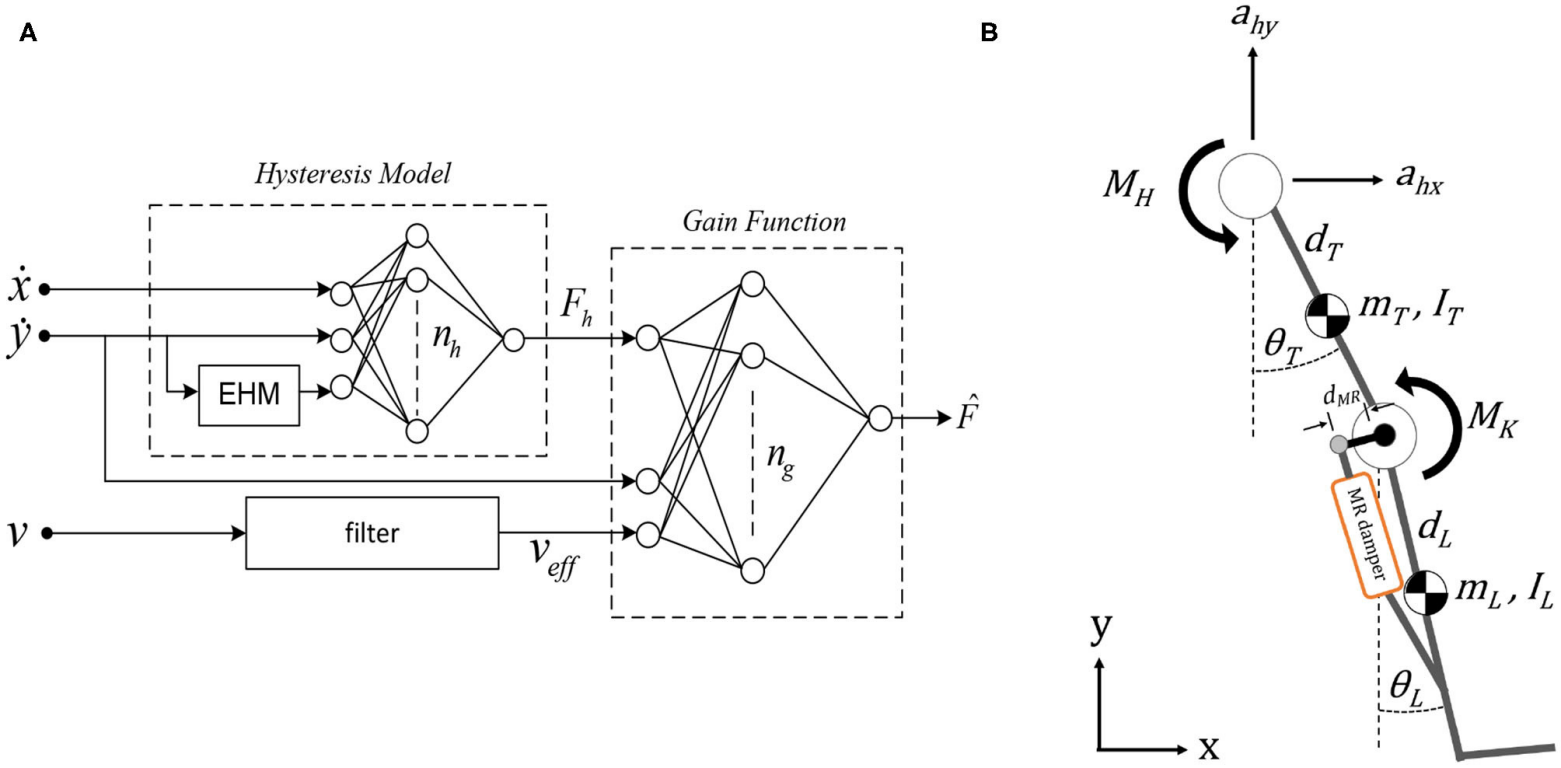
**(A)** Control structure of magnetorheological (MR) damper (Ekkachai et al., 2013). **(B)** Double pendulum model to simulate swing phase with MR damper attachment with distance *d*_*MR*_ from the knee joint.

In this study, prosthetic knee is actuated by MR damper having non-linear characteristics such as hysteresis and dynamic response that are difficult to control. To capture these behaviors of MR damper, the elementary hysteresis model (EHM) based feed-forward neural network (FNN) model is used in our simulation. It was proposed in Ekkachai et al. (2012) and modified in Ekkachai and Nilkhamhang (2016). The model consists of two FNNs. Here, one FNN coupled with EHM acted as a hysteresis model, and the output of this network was fed to the other FNN that acted as the gain function. Voltage is filtered by the first-order lag filter. Piston velocity and acceleration are used as inputs to estimate MR damper force. The MR damper model is shown in Figure 1A. The model was trained by using data from the experimental system of an actual MR damper, Lord RD-8040-1, described in Ekkachai et al. (2013).

(1) $$\begin{array}{l} M_{K}=d_{MR}\cdot |\hat{F}|\text{cos }\theta_{K} \end{array}$$

The MR damper is attached at a distance, *d*_*MR*_, away from the knee joint. Based on this distance, the torque generated at knee joint by the MR damper is calculated by Equation (1), where $\hat{F}$ is the force generated by MR damper (Figure 1A) and θ_*K*_ is the knee angle. θ_*K*_ is calculated by θ_*K*_ = θ_*T*_ − θ_*L*_, where subscripts T and L denote thigh and leg segment, respectively, as shown in Figure 1B.

#### 2.1.2. Environment Model

The double pendulum model is proposed as the environment model for the swing phase (Putnam, 1991). The model consists of two links, that is, thigh and a lumped shank, as well as a foot segment, as depicted in Figure 1B. There are two actuated joints with a total of four degrees of freedom, where the hip joint has one rotational degree of freedom on the z-axis and two translation degrees of freedom on the *x* and *y*-axes; meanwhile, the knee joint has one rotational degree of freedom on the *z*-axis.

(2) $$\begin{array}{l} M_{K}=I_{L}\alpha_{L}+m_{L}d_{L}(L_{T}\alpha_{T}\text{cos}(\theta_{L}-\theta_{T})+\omega_{T}L_{T}\text{sin}(\theta_{L}-\theta_{T})\\ \;+a_{hx}\text{cos }\theta_{L}+(a_{hy}+g)\text{sin }\theta_{L}) \end{array}$$

(3) $$\begin{array}{l} M_{H}=M_{K}+(m_{L}L^{2}{\text{​}}_{T}+I_{T})\alpha_{T}+m_{L}d_{L}L_{T}(\alpha_{L}\text{cos}(\theta_{L}-\theta_{T})\\ \;-\omega^{2}{\text{​}}_{L}\text{sin}(\theta_{L}-\theta_{T}))+(m_{L}L_{T}+m_{T}d_{T})(a_{hx}\text{cos }\theta_{T}\\ \;+(a_{hy}+g)\text{sin }\theta_{T}) \end{array}$$

This model was simulated in MATLAB (Mathworks Inc., Natick, MA, USA) SimMechanics environment. The torque generated by each joint, derived from Lagrange equation, are governed by Equations (2) and (3), where *M*_*K*_ and *M*_*H*_ are the torques at knee and hip, respectively. *m, I, d, and L* are segment mass, moment of inertia at segment's center of mass, length measured from the proximal end of the segment to the center of mass, and segment length, respectively. The subscripts *L* and *T* denote the leg segment and thigh segment, respectively, while *a*_*hx*_ and *a*_*hy*_ are the linear acceleration at hip joint along the *x* and *y* axes. Further, θ, ω, α, *and g* are the angle, angular velocity, angular acceleration, and gravitational constant at 9.8 *m*/*s*^2^, respectively.

#### 2.1.3. Dataset

**Figure 2:**
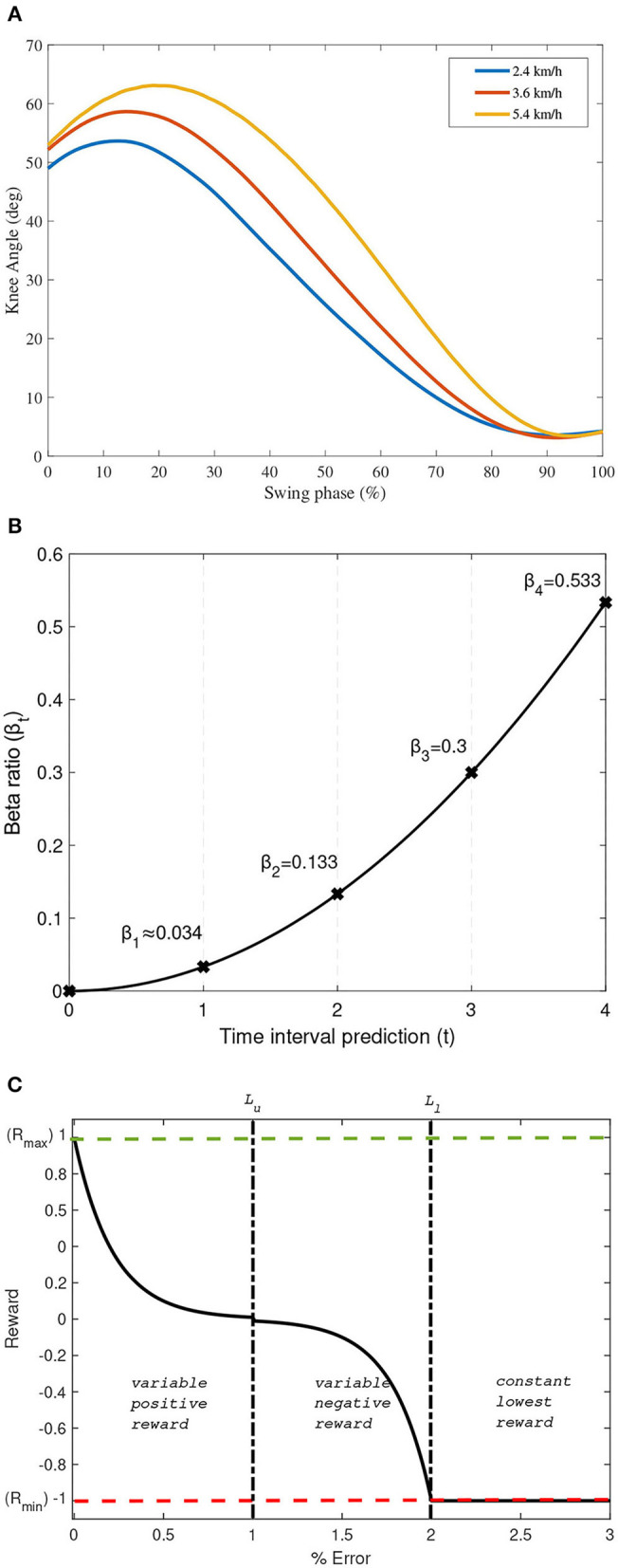
**(A)** Average knee angle data used in this study. **(B)** β_*t*_ as an exponential function with *n* = 4. **(C)** Proposed reward shaping function as a function of *E*_*t*_.

The gait data used in this study are also normal gait data collected from Ekkachai and Nilkhamhang (2016) for convenience in comparison study of the controller. In this manner, the proposed controller performance can be compared to the previous method with same dataset. It allows us to analyze the difference from the previous work result keeping the same experimental condition. A male subject with 83 kg of weight and 1.75 m height at the time of the experiment were asked to walk on a treadmill at various speed, where in this study walking speed was set at 2.4, 3.6, and 5.4 km/h (Ekkachai and Nilkhamhang, 2016). A high-speed camera was used to capture joints coordinate and later converted to relative joint angles. To capture the respective joints coordinate, reflective markers were placed at hip, knee, and ankle joints. In this study, as only the control in the swing phase is discussed, the gait data used will be constrained into the swing phase only. Since we proposed a RL-based algorithm, all the recorded knee angle data with a total of 200 sets per walking speed will be used. The average knee angle data at the swing phase used in this study are depicted in Figure 2A.

### 2.2. Q-Learning Control

Here, the proposed Q-learning control is discussed. Q-learning belongs to the tabular RL group in the machine learning algorithm. Generally, RL learns the control policies within a specified environment where the performance and training information are provided in terms of whether the applied control policy is a success or failure (Sutton and Barto, 2018). Success or failure in this case is determined by a certain performance index depending on the system and environment involved.

#### 2.2.1. Q-Learning Structure

The general structure of RL is consisted of an agent and a system/environment. An agent executes an action, *a*_*t*_, to the system and environment. Based on the given action, the system will react to another state, *s*_*t*_, while also gives a reward, *R*_*t*_, based on the performance index calculated from the current state. In this study, the agent is the *Q*-function with a mathematical description, as shown in Equation (4).

(4) $$\begin{array}{l} Q_{\left(s_{t},a_{t}\right)}\leftarrow Q_{\left(s_{t},a_{t}\right)}+\alpha \left[R_{\left(s_{t},a_{t}\right)}+\gamma \text{ max }Q_{\left(s_{t+1},a_{t}\right)}-Q_{\left(s_{t},a_{t}\right)}\right] \end{array}$$

In Equation (4), *Q* and *R* are the action-value and reward functions, respectively. Further, *s, a*, α, *and γ* are the state, action, learning rate, and discounted rate, respectively, while subscript *t* denotes the time. Learning rate and discounted rate are dimensionless variables between 0 and 1. Higher learning rate, which if sets closer to 1, indicates that the *Q*-function is updated quickly per iteration, while the *Q*-function is never be updated if it is set to 0. The discounted factor is a variable that determines how the *Q*-function acts toward the reward. If it is set closer to 0 means, it will only consider the instantaneous reward, while if it is set closer to 1, it strives more into the long-term higher rewards (Sutton and Barto, 2018).

(5) $$\begin{array}{l} Q_{\left(\theta_{K\left(t\right)},\dot{\theta_{K\left(t\right)}},a_{t}\right)}\leftarrow Q_{\left(\theta_{K\left(t\right)},\dot{\theta_{K\left(t\right)}},a_{t}\right)}+\alpha [R_{t}+\gamma \text{ max }Q_{\left(\theta_{K\left(t+1\right)},\dot{\theta_{K\left(t+1\right)}},a_{t}\right)}\\ \begin{array}{l} \;-Q_{\left(\theta_{K\left(t\right)},\dot{\theta_{K\left(t\right)}},a_{t}\right)}] \end{array} \end{array}$$

In this study, Q-learning is proposed to be used as a controller of a dynamics system of the MR damper in the prosthetic knee in a double pendulum-simulated environment. The state is the parameter extracted from the environment that contains necessary information to be used to evaluate the control policies. In most cases, *Q*-function with multistate is used to better learn the environment (Fernandez-Gauna et al., 2013; Sadhu and Konar, 2017; Chai and Hayashibe, 2020). Particularly, this paper (Chai and Hayashibe, 2020) has explored deep RL for motion generation in a simulated environment. In this study, θ_*K*_ and derivative of knee angle, $\dot{\theta_{K}}$, are used as states, while the command voltage, *v*, is used as the action. Thus, the update rule of the *Q*-function can be written as in Equation (5). As Q-learning is following an off-policy method, actions were selected based on the maximum value of the Q-function on the current states, max*Q*_(*s*_1(*t*)_, *s*_2(*t*)_)_. Meanwhile at the initialization stage of learning, action selection follows a greedy policy to explore the Q-function for possible solutions.

(6) $$\begin{array}{l} Q_{\left(\theta_{K\left(t\right)},\dot{\theta_{K\left(t\right)}},a_{t}\right)}\leftarrow Q_{\left(\theta_{K\left(t\right)},\dot{\theta_{K\left(t\right)}},a_{t}\right)}+\alpha [\overset{n}{\underset{t=1}{\sum }}\beta_{t}R_{t}\\ \begin{array}{l} \;+\gamma \text{ max }Q_{\left(\theta_{K\left(t+1\right)},\dot{\theta_{K\left(t+1\right)}},a_{t}\right)}-Q_{\left(\theta_{K\left(t\right)},\dot{\theta_{K\left(t\right)}},a_{t}\right)}] \end{array} \end{array}$$

#### 2.2.2. Reward Shaping Function

The structure of the reward mechanism in the Q-learning algorithm used in this study is modified into a rationed multiple rewards as a function of time. This structure enables the learning process to provide more reward to latter horizon events due to the response time required by the MR damper to generate the necessary damping mechanism. The mathematical descriptions of this multiple reward mechanism are expressed in Equation (6), where $\beta_{t}=ct^{2}$ and $\overset{n}{\underset{t=1}{\sum }}\beta_{t}=1$.

In Equation (6), β_*t*_ is the specifically designed ratio of reward priority, *n* is the number of prediction horizon, and *c* is a constant that depends on *n*. In this study, *n* is set to 4; thus, *c* = 0.033 to be conveniently compared to the NNPC algorithm studied in Ekkachai and Nilkhamhang (2016) that set the prediction horizon to 4. Further, the reward priority given at the specified prediction horizon is an exponential function, as depicted in Figure 2B.

As the controller aims to mimic the biological knee trajectory in the swing phase, the reward will be given according to whether the prosthetic knee can follow the biological knee trajectory. In this study, the reward is designed as a function of a performance index (*PI*). A simple absolute error, *e*_*t*_, is selected as the performance index and evaluated per interval time. The reward function is also designed to have a continuous value over a specified boundary and follow a decaying exponential function. The mathematical descriptions of the proposed designed reward functions are expressed in Equations (7)–(11).

(7) $$\begin{array}{l} R_{t}=f\left(PI\right) \end{array}$$

(8) $$\begin{array}{l} PI=e_{t}=\left|\frac{\theta_{K}-\theta_{K\left(val\right)}}{\theta_{K\left(val\right)}}\right| \end{array}$$

(9) $$\begin{array}{l} R_{max}\delta^{E_{t}}\;;\;0<E_{t}<L_{u} \end{array}$$

(10) $$\begin{array}{l} R_{t}=\{R_{min}\delta^{\left|L_{t}-E_{t}\right|}\;;\;L_{u}<E_{t}<L_{l} \end{array}$$

(11) $$\begin{array}{l} R_{min}\;;\;E_{t}>L_{l} \end{array}$$

In Equations (7)–(11), θ_*K*(*val*)_ is the validation of knee angle at time *t*, *R*_*max*_, and *R*_*min*_ are the maximum reward and minimum reward set to 1 and −1, respectively. *E*_*t*_ is the percentage of *e*_*t*_, which can be written as *E*_*t*_ = 100*e*_*t*_. Further, δ, *L*_*u*_, *and L*_*l*_ are the reward constants set arbitrarily to 0.01, performance limit to obtain the positive reward, and performance limit to obtain the lowest reward, respectively. In this study, *PI* is aimed to be within 0.01, indicating that the error should be under 1%. Thus, *L*_*u*_ is set to be 1, and *L*_*l*_ could be set to any number larger than *L*_*u*_ to provide a variable negative reward. In this case, *L*_*l*_ is set to be twice the value of *L*_*u*_.

The graphical description of this reward design is depicted in Figure 2C. Note that δ, *L*_*u*_, *L*_*l*_, *R*_*max*_, and *R*_*min*_ can be defined accordingly for other applications depending on the system being evaluated. The reward shaping function is preferred to follow a decayed exponential function rather than a linear function to better train the *Q*-function to reach the state with the largest reward value, which can lead to faster convergence.

## 3. Simulation and Results

**Figure 3:**
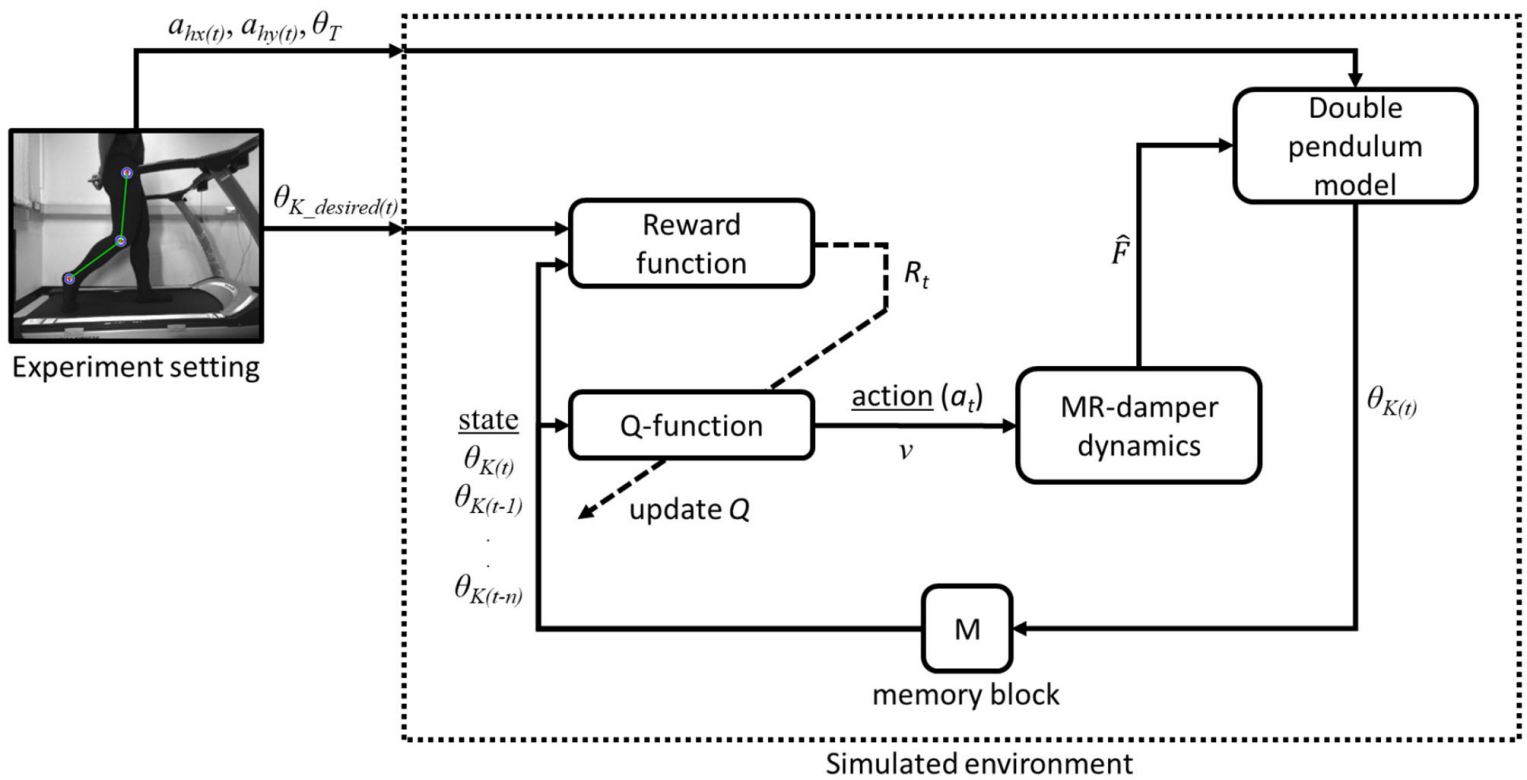
Block diagram of the proposed Q-learning control in a simulated environment with external experiment data.

In this section, a simulation of swing phase control using the proposed controller is discussed along with a comparison study. The simulation was computed using IntelⓇ Core^*TM*^ i7 6th Generation 3.5 GHz processor with 8 GB RAM. The overall diagram of our study is depicted in Figure 3. The figure shows an experiment setting that provide kinematics data of the subject and a simulated environment where our proposed framework is tested. On the simulated environment, we have a *Q*-function block with input of multistate of knee angle from double pendulum model and updated by the reward function. The input of the reward function are the actual knee angle θ_*K*_(*t*) and the desired knee angle θ_*K*(desired)_(*t*) from experimental data. The output of *Q*-function is an action (*a*_*t*_) in the form of control voltage (*v*) that is passed on to MR-damper dynamics block. The voltage is converted into $\hat{F}$ following Figure 1A and passed on to the double pendulum model for swing phase simulation.

There are several parameters in Q-learning control that must be defined and optimized. First, as this control approach is a tabular RL using the Q-learning method, each value of the *Q*-function is stored in a Q-matrix. The size of the Q-matrix depends on the number of states and actions. In this simulation, the structure of the Q-matrix is a three-dimensional matrix consisting of *l* rows of state θ_*K*(*t*)_, *m* columns of state $\dot{\theta_{K\left(t\right)}}$, and *n* layers of action *v*. Q-matrix must cover all the states and actions available on the system. Based on the data used, the state θ_*K*(*t*)_ is within the range of 0 and 70° with a predefined step size of 0.5°, resulting with 141 rows. State $\dot{\theta_{K\left(t\right)}}$ is set from −7 to 7° per unit of time with predefined 0.05 step size, thus resulting with 281 columns. The range of command voltage is set from 0 to 5 V with 0.1 resolution, resulting with 51 layers of action.

Second, learning rate α need to be defined. In this simulation, several values of learning rate are simulated to determine its effect on the number of iteration required to achieve best performance. The performance index used to evaluate this simulation is the normalized root mean squared error (*NRMSE*) as expressed in Equation (12), where *n*_*s*_ is the number of samples in dataset.

(12) $$\begin{array}{l} NRMSE=\frac{\sqrt{\frac{1}{n_{s}}\sum_{t=1}^{n_{s}}{\left[\theta_{K\left(\text{desired}\right)}\left(t\right)-\theta_{K}\left(t\right)\right]}^{2}}}{\text{max}\left(\theta_{K\left(\text{desired}\right)}\right)-\text{min}\left(\theta_{K\left(\text{desired}\right)}\right)} \end{array}$$

**Figure 4:**
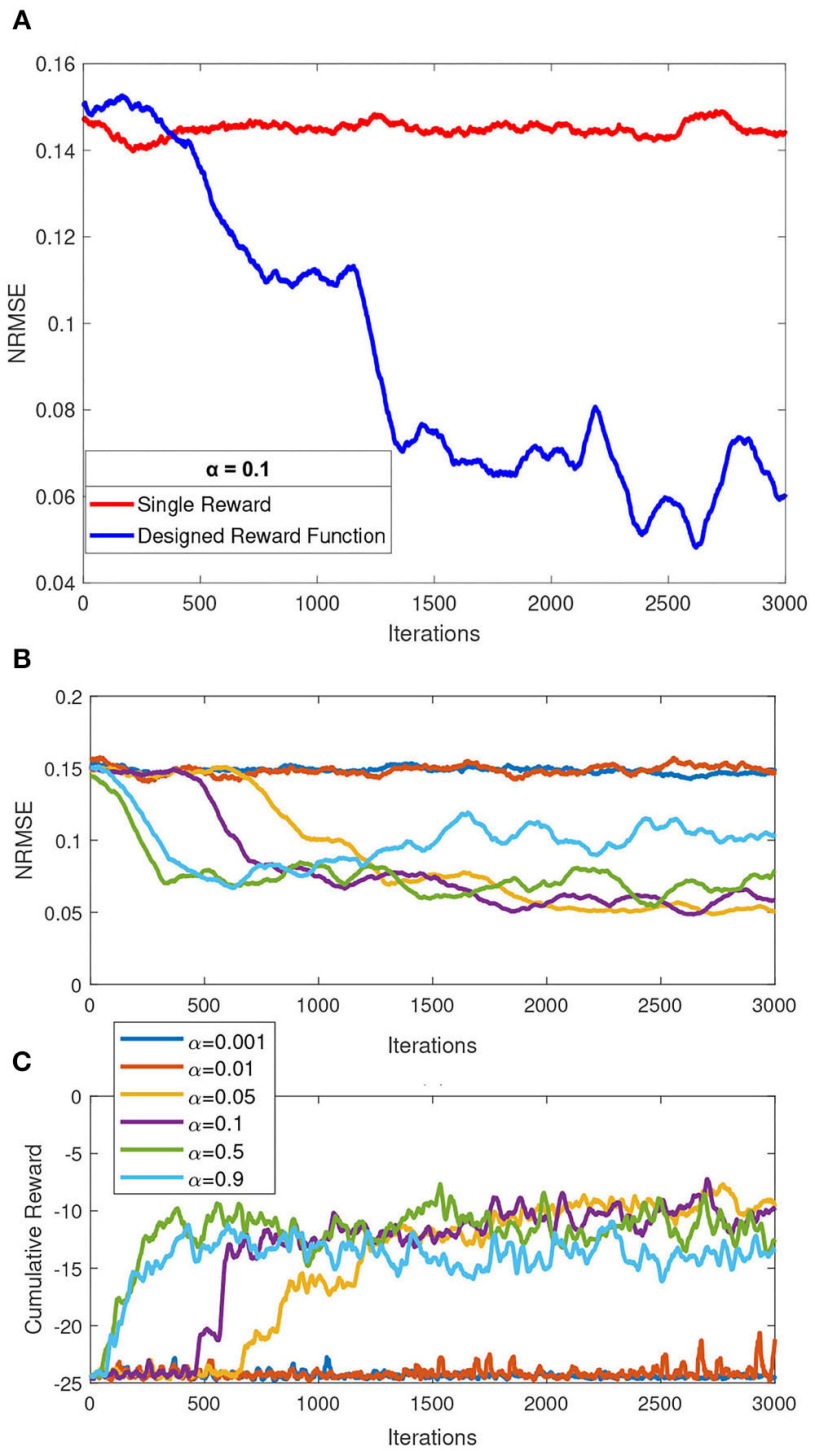
Summary of simulation results over a constrained iteration of 3000. **(A)** Comparison of single reward mechanism and our proposed reward shaping function. **(B)** Effect of various learning rates to the overall performance (normalized root mean squared error, NRMSE). **(C)** Comparison of cumulative reward over iteration by each of the simulated learning rates.

On the first simulation, we compared our reward shaping function as formulated in Equations (7)–(11) to a single reward mechanism expressed in Equation (4). We used 2.4, 3.6, and 5.4 km/h walking speed dataset, simulated separately with same value of randomized Q-matrix initialization. We then measured the moving average of *NRMSE* parameter with a constrained maximum iterations of 3000 and a fixed learning rate of 0.1. The results of this simulation are depicted in Figure 4A. It can be concluded from this simulation that the reward shaping function performed better over time in terms of *NRMSE*, compared to a single reward function.

In the second simulation, several values of learning rate α = [0.001, 0.01, 0.05, 0.1, 0.5, 0.9] are picked a priori to be simulated with a maximum 3000 iteration in a single speed simulation (mid speed of 3.6 km/h). For each learning rate, simulation was performed three times and average *NRMSE* for each learning rate were recorded. The effect of these learning rate to *NRMSE* is shown in Figures 4B,C. We concluded that the two lowest learning rate (α = 0.001 and α = 0.01) simulated with a constrained iteration of 3,000 performed the worst among other learning rates. Those two learning rates did also not show any significant performance changes over the constrained iteration. As also observed, a higher learning rate does not guarantee better performance, as inspected from α = 0.9, compared to α = [0.05, 0.1, 0.5]. For the next simulation, we picked learning rate α = 0.5 based on this simulation and considering faster exploration of Q-matrix that could potentially lead to finding better local minimum as solution.

There are many approaches to train the *Q*-function in this study. Training one *Q*-function for a specific case of a single walking speed is easy, while training multispeed at once under one *Q*-function is challenging. In this simulation, training multispeed under one control policy is proposed. Slowest, mid, and fast walking speeds of 2.4, 3.6, and 5.4 km/h, respectively, are used for training. In this simulation, the time interval is set to 20 ms; thus, the action or command voltage to the prosthetic knee is updated every 20 ms. The dataset of 2.4, 5.4, and 3.6 km/h is selected randomly for every iteration of the simulation. There are two conditions for the simulation to stop: first is if all the *NRMSE* of all trained speed falls under the defined *PI* criterion, and second is if all the trained speed converges into one final value of *NRMSE* for at least after 10 further iterations.

**Figure 5:**
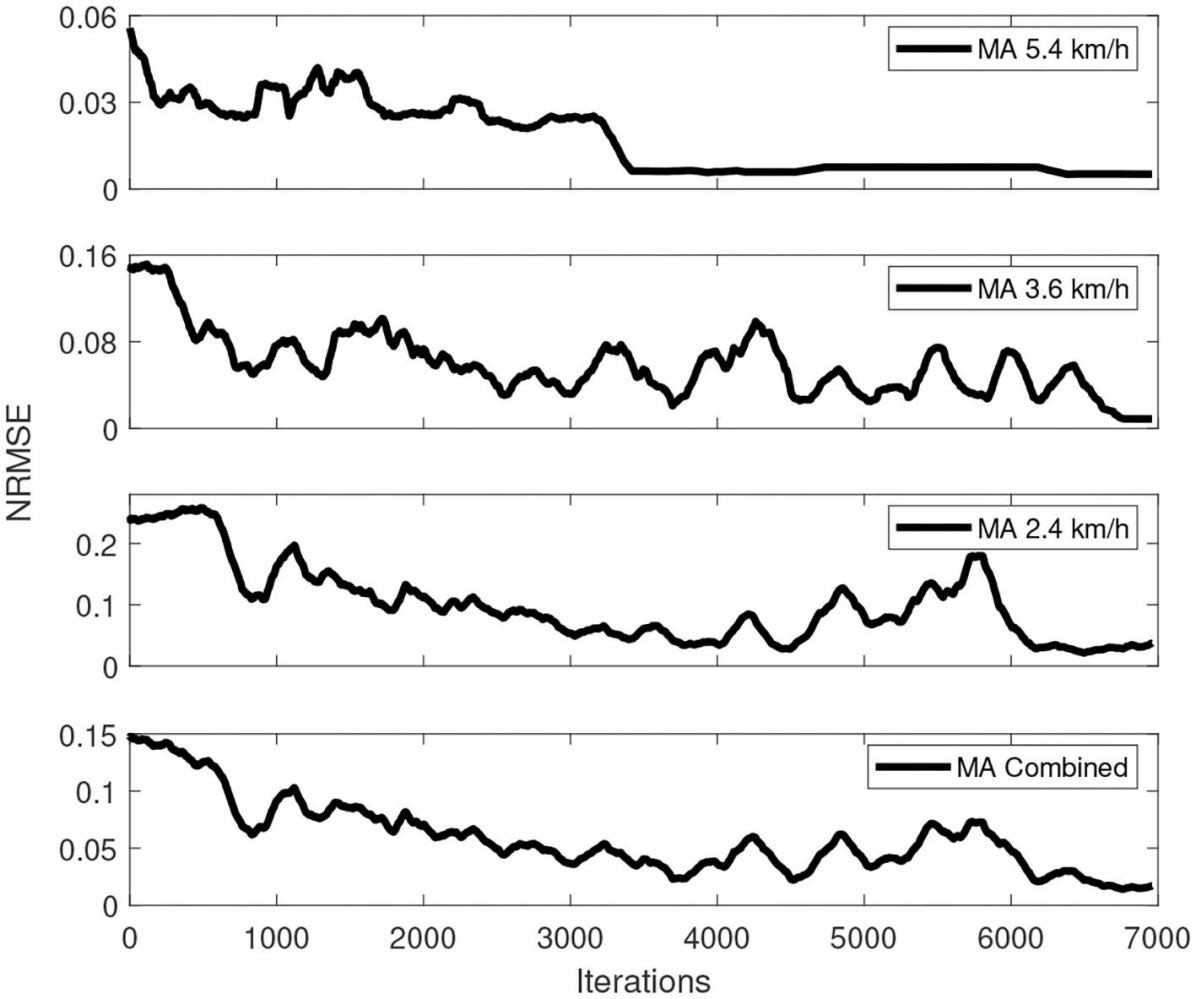
Overall training process of multispeed of walking under one control policy simulation.

The best training process of this simulation over a total of 10 training processes is depicted in Figure 5. As shown in this figure, the fastest convergence was achieved by the fastest walking speed, which converges at around 3,300 iterations, followed by the walking speed of 3.6 km/h, which converges at around 6,700 iterations, and the latest is the slowest walking speed, which converges at around 6,900 iterations. This occurrence happened because a faster walking speed generally indicates a short time in the gait cycle, resulting in a less swing-phase time. The lesser time in the swing phase with a fixed control interval of 20 ms indicates that the *Q*-function calculates fewer actions than the slower walking speed dataset.

## 4. Discussion

In this study, we investigated our proposed control algorithm for the swing phase controller in the MR-damper-based prosthetic knee. The proposed controller was designed with the structure of a tabular reinforcement Q-learning algorithm, a subset in machine learning algorithms. The Q-learning control comprised a *Q*-function that stores its value in a Q-matrix and a reward function following the reward shaping function proposed in this study. The advantages of using this control structure are that it can be trained online, and also it is a model-free control algorithm that does not require prior knowledge of the system to be controlled. A variable reward as a function of *PI* associating a decayed function, which is proposed as a reward function herein, has led to a better reward mechanism. We have shown that our proposed reward function demonstrated a trend of faster convergence compared to a single reward mechanism as depicted in Figure 4A.

**Figure 6:**
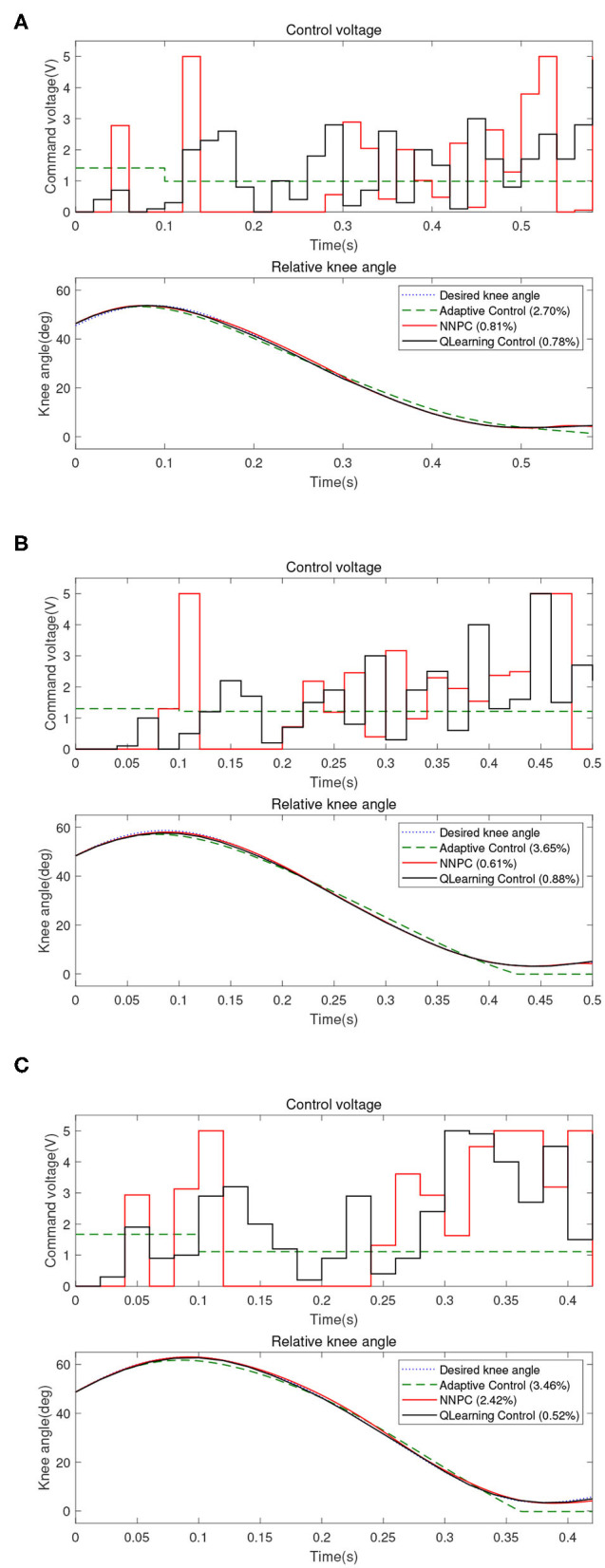
Comparison between user-adaptive control (green dashed line), neural network predictive control (NNPC) (red line), and Q-learning control (black line) for different walking speeds: **(A)** 2.4 km/h, **(B)** 3.6 km/h, and **(C)** 5.4 km/h.

The proposed controller is then compared to the user-adaptive controller (Herr and Wilkenfeld, 2003) and the NNPC algorithm (Ekkachai and Nilkhamhang, 2016). The comparison of 2.4, 3.6, and 5.4 km/h walking speed are depicted in Figure 6 and Table 1. The table depicts that for the walking speed of 2.4 km/h, Q-learning method performed the best with 0.78 of *NRMSE*, compared to NNPC (0.81) and user-adaptive control (2.70). Further, for the walking speed of 3.6 km/h, the best performance was achieved by NNPC with 0.61 of *NRMSE*, compared with Q-learning (0.88) and user-adaptive control (3.65). Lastly, for the walking speed of 5.4 km/h, Q-learning performed the best with the lowest *NRMSE* of 0.52, compared with NNPC (2.42) and user-adaptive control (3.46). Overall, Q-learning method perform within 1% of *NRMSE*, which followed the designed common reward function for different walking speed.

**Table 1:** Comparison between user adaptive, neural network predictive control (NNPC), and Q-learning control.

**Walking speed (km/h)**	**NRMSE(%)**		
	**User-adaptive**	**NNPC**	**Q-learning**
2.4	2.70	0.81	0.78^*^
3.6	3.65	0.61^*^	0.88
5.4	3.46	2.42	0.52^*^
Average	3.27	1.28	0.73^*^

* *Best performance*.

This control structure also shows adaptability to various walking speeds. Moreover, we have successfully trained a unified control policy for every simulated walking speed. *PI* verified with the experimental result indicates that this control structure performs better than the user-adaptive control. Moreover, in some of the walking speeds, this control structure performs better than the NNPC algorithm. The total performance over different walking speeds showed promising results by using the proposed approach.

In terms of cost function, knee trajectory is only one of the parameters to be optimized among other correlated systems, such as ankle and foot prostheses, to achieve better gait symmetry and reduce metabolic costs. Although there has not been a detailed study about the acceptable criterion in terms of the *NRMSE* performance index of the knee trajectory in a prosthetic knee, this study aims to mimic the biological knee trajectory, which is shown by *PI*.

On the applicability point of view, our proposed Q-learning control had no prior knowledge of the structure and characteristics of MR-damper. Signals observed by Q-learning control were the states of knee angle and its derivatives, as well as the reward signal *R*_*t*_ that was given based on the performance of the controller to shape the control policy. Based on this facts, our proposed Q-learning control can potentially be used for other structure of MR-damper or even other impedance-based machine for semi-active prosthetic.

Although we cannot provide detailed comparison of our proposed method with another RL-based method in Wen et al. (2019), a brief comparison is discussed as follows. The ADP-based RL algorithm resulted in 2.5° of *RMSE* on the robotic knee kinematics. The average performance of our proposed method was 0.73 of *NRMSE* or was 1.59° if converted to average *RMSE*. Conversely, in this study, we employed the RL algorithm to control the output of the control voltage for the MR damper, resulting in only one simple output variable. Meanwhile, this existing study (Wen et al., 2019) used the RL algorithm to tune a total of 12 impedance parameters of the robotic knee; thus, the output variables are 12. We also treated the swing phase as one state, while in Wen et al. (2019), the swing phase was divided into swing flexion and swing extension where the ADP tuner would tune the impedance parameters accordingly with respect to each state.

In this study, we focused on developing a unique control that can adapt and accommodate a range of subject-specific walking speed. Unique means that it can only be valid for the subject. The reason was, like any other prosthetic, it is tuned personally to the wearer. In this study, the control policy that we train is valid only for the subject whose data we used. However, the idea of our proposed control framework and algorithm can be applied to other subjects.

While it has shown a promising result, we also identified some of the limitations of our study. Using the computational hardware mentioned at the previous section and source code implemented in MATLAB, the overall calculation and online update *Q*-function process consumed approximately 40.4 ms, while each evaluation of NNPC with pretrained swing phase model consumed approximately 13.2 ms (Ekkachai and Nilkhamhang, 2016). Changing the source code implementation in C language and using dedicated processing hardware could shorten the calculation time to be within the proposed control interval of 20 ms.

There are several areas that can be explored for future works. First, another training strategy can be explored further to shorten the calculation time. Second, this study proposed a tabular-discretized *Q*-function stored in a Q-matrix. A continuous *Q*-function could also be explored to better cover all the states and actions. Third is to test our proposed control strategy to other subjects and possibly to test a transfer learning approach from control policy that was learnt in this study for dataset from other subjects.

## Data Availability Statement

The data analyzed in this study is subject to the following licenses/restrictions: datasets analyzed in this article are available upon request. Requests to access these datasets should be directed to kittipong.ekkachai@nectec.or.th.

## Ethics Statement

Ethical review and approval was not required for the study on human participants in accordance with the local legislation and institutional requirements. The patients/participants provided their written informed consent to participate in this study.

## Author Contributions

YH contributed to algorithm design and development, data analysis and interpretation, and writing the first draft. KE supported the development of the system and environment model, collecting datasets, and data analysis. MH provided critical review and contributed additional texts to the draft. WK contributed to study conception and design, provided critical review, and supervised the overall study. All authors read, reviewed, and approved the final manuscript.

## Conflict of Interest

The authors declare that the research was conducted in the absence of any commercial or financial relationships that could be construed as a potential conflict of interest.

## Footnotes

^1^Available online at: https://www.ossur.com/en-us/prosthetics/knees/power-knee (accessed July 17, 2020).
